# Progress in Disease of Digestive Organs

**Published:** 1901-07-13

**Authors:** 


### PROGRESS IN DISEASE OF DIGESTIVE ORGANS.

(*Continued from page* 198.)

**Dysentery.**—Flexner[34] recently studied the disease in the Philippines and found two distinct forms, one due to a bacillus and the other to the amœba. The acute form was produced by a bacillus identical with that discovered by Shiga in Japan, which seems nearly allied to B. typhosus. Chronic forms in the tropics are commonly amœbic. Some success was obtained in bacillary dysentery by vaccination with dead cultures.

Lauder Brunton,[35] in speaking of chronic diarrhœas, refers to the value of the new preparations of tannin which are not soluble in the stomach, but reserve their activity for the intestine. Copper sulphate in pills of half a grain to a grain, and above all castor oil in doses of 10 to 60 minims daily, are often most useful. J. P. Parkinson[36] reports favourably of Honthin, a compound of tannin, albumen, and keratin, of which only 28 per cent. is dissolved in the stomach. He has found it very beneficial in the diarrhœa of children and in chronic colitis, and it seems worthy of trial in cases of dysentery. In amœbic dysentery Aderhold[37] advises daily enemata given with the hips raised a foot or more. The best formulæ seem to be quinine sulphate 1 in 5,000 up to 1 in 1,000, perchloride of mercury 1 in 10,000 to 1 in 5,000, hydrogen peroxide 1 in 20 to 1 in 5, and methylene blue 1 in 100 to 1 in 1,000. Various forms may be used alternately with advantage.

W. J. Buchanan[38] reports from Bengal on the treatment with salines of 300 more cases, making a total of 855, with a mortality of little over 1 per cent. The disease is most prevalent in the rainy season and the mortality among native patients is usually about 33 per cent. In Indian gaols the mortality in 60,000 cases of late years has been only 7 per cent. Under the writer's method with prompt treatment in a hospital, this has been again reduced. The drug used was sodium sulphate, a drachm in fennel water being given from four to eight times in a day. A stool next morning was inspected and unless every trace of blood and mucus had disappeared the remedy was continued. In a few days success was generally obtained. However, the method must be used only for acute, and not for chronic or relapsing cases with ulceration of the colon. Here an occasional dose may be given, but bismuth, salol, with sometimes a dose of castor and laudanum, anthelmintics, and a diet of boiled milk and rice water should be tried. R. A. Mate,[39] in the Natal campaign found sulphate of magnesia disappointing, but he had great success where he added hydrarg. perchloride, opium, and a grain of quinine to the drachm doses of magnesia which were given every three hours. Irrigation at the same time with boric solution, and the use of turpentine stupes were productive of much relief. Mott and Barham,[40] in their report on the ulcerative colitis of asylums under the London County Council, think that it is ordinary dysentery, and that sufficient care is not taken in cleansing and disinfecting mattresses and other articles, and that the dormitories are overcrowded. The disease appears to be constantly present in certain asylums, and the state of affairs is very serious.

**Cirrhosis of Liver.**—The cardiac form is discussed by Piery,[41] who gives reason to think that it is not due to mere venous engorgement, though favoured by it. Alcohol, intestinal toxins, and those of rheumatism and tuberculosis are the active agents. These views are supported by microscopical and experimental evidence. W. F. Ford[42] reviews the subject of cirrhosis produced by *biliary obstruction.* Mangelsdorff found 184 cases reported, and the writer adds 24 more up to the present time. The causes are gall stones, enlarged glands, malignant growths, and possibly congenital deficiency of the ducts, though in the last case it may be that the cirrhosis is due to a poison circulating through the fœtal liver and not to the retention of the bile. Complete obstruction in animals produces after a sufficient time interlobular cirrhosis of considerable extent, as shown by the experiments of Harley and others. In man such obstruction is followed by cirrhosis, which is due, however, in part to the inflammation which always accompanies obstruction. This latter cirrhosis cannot be distinguished anatomically from that seen in Hanot's hypertrophic biliary cirrhosis. The symptoms, however, of obstructive cirrhosis are very different. The course of the disease is acute, the loss of weight and emaciation are rapid, there are no periods of intermission, and though any fever is rare, vomiting is common. The jaundice, instead of being slight at first but increasing, is deep at the commencement. The stools are always clay coloured, and ascites, œdema, and enlarged abdominal veins are often found, unlike what is seen in Hanot's cirrhosis. Thus obstructive cirrhosis has a marked character of its own. *Congenital obliteration* of ducts with cirrhosis has only been reported in some 60 instances, most of which were collected by J. Thomson. H. D. Rolleston[43] mentions a recent case. The patient died at the age of six months with jaundice dating from birth and an enlarged liver and spleen. The cirrhosis was mixed, monolobular and multilobular, and the common and cystic ducts were represented by fibrous cords. It is probable that irritative bodies in the fœtal blood akin to toluylendiamine have passed partly by the umbilical vein into the liver direct, causing multilobular cirrhosis, and partly by the ductus venosus and hepatic artery, causing monolobular cirrhosis and cholangitis. Both forms of cirrhosis are present together in some of these cases, and the obliteration of the ducts is probably not due to failure of development, but a sequel to the cholangitis occurring in the extremely narrow ducts of infants. It is a curious fact that several cases of this disease may occur in the same family. T. E. Blomfield[44] reports the deaths of two brothers from this cause, and the mother stated that her two previous children were similarly affected. She had suffered from some gastric trouble during her pregnancies, and the theory that the disease is due to the absorption of some intestinal toxin by the mother is supported by other instances. One woman lost seven children in this way, and no definite connection with syphilis seems made out. Browiez[45] argues that all forms of jaundice are due to the excessive activity of hepatic cells. When these cells are abnormal they cannot empty the excess of bile into the intercellular canals. Mechanical factors act by disturbing the circulation in the capillaries.

[34] Pract., Feb., p. 223. [35] Clin. Jour., April 21. [36] Lancet, Dec. 22. [37] Brit. Med. Jour., Jan. 19. [38] Brit. Med. Jour., April 13. [39] Brit. Med. Jour., May 11. [40] Brit. Med. Jour., April 6. [41] Amer. Jour. Med. Sci., Feb. [42] Amer. Jour. Med. Sci., Jan. [43] Brit. Med. Jour., March 30. [44] Brit. Med. Jour., May 11. [45] Amer. Jour. Med. Sci., Feb.

